# Anaemia and Iron Homeostasis in a Cohort of HIV-Infected Patients: A Cross-Sectional Study in Ghana

**DOI:** 10.1155/2016/1623094

**Published:** 2016-03-22

**Authors:** Christian Obirikorang, Razak Gyesi Issahaku, Derick Nii Mensah Osakunor, James Osei-Yeboah

**Affiliations:** ^1^Department of Molecular Medicine, School of Medical Sciences, College of Health Sciences, Kwame Nkrumah University of Science and Technology, Kumasi, Ghana; ^2^Medical Laboratory Department, Tamale Teaching Hospital, Tamale, Ghana; ^3^Department of Medical Laboratory Sciences, University of Health and Allied Sciences, Ho, Ghana

## Abstract

*Aim.* We determined the prevalence of anaemia and evaluated markers of iron homeostasis in a cohort of HIV patients.* Methods.* A comparative cross-sectional study on 319 participants was carried out at the Tamale Teaching Hospital from July 2013 to December 2013, 219 patients on HAART (designated On-HAART) and 100 HAART-naive patients. Data gathered include sociodemography, clinical history, and selected laboratory assays.* Results.* Prevalence of anaemia was 23.8%. On-HAART participants had higher CD4/CD3 lymphocyte counts, Hb, HCT/PCV, MCV, MCH, iron, ferritin, and TSAT (*P* < 0.05). Hb, iron, ferritin, and TSAT decreased from grade 1 to grade 3 anaemia and CD4/CD3 lymphocyte count was lowest in grade 3 anaemia (*P* < 0.05). Iron (*P* = 0.0072) decreased with disease severity whilst transferrin (*P* = 0.0143) and TIBC (*P* = 0.0143) increased with disease severity. Seventy-six (23.8%) participants fulfilled the criteria for anaemia, 86 (26.9%) for iron deficiency, 41 (12.8%) for iron deficiency anaemia, and 17 (5.3%) for iron overload. The frequency of anaemia was higher amongst participants not on HAART (OR 2.6 for grade 1 anaemia; OR 3.0 for grade 3 anaemia).* Conclusion.* In this study population, HIV-associated anaemia is common and is related to HAART status and disease progression. HIV itself is the most important cause of anaemia and treatment of HIV should be a priority compared to iron supplementation.

## 1. Introduction

Iron deficiency is the leading cause of anaemia in the developing world [1]; the World Health Organization (WHO) and World Bank have ranked iron deficiency anaemia as the third leading cause of Disability-Adjusted Life Years (DALYs) lost for females (15–44 years) and as part of top 10 disease burdens for men [2]. The condition has also been classified as a severe public health problem in children and for pregnant women in more than 67 countries [3].

At the end of 2012, an estimated 35.3 million people worldwide were living with HIV and Sub-Saharan Africa was the region most heavily affected [4, 5]. Anaemia is a common feature of HIV infection (20–80%) [6] and occurs in about 35% of patients who initiate Highly Active Antiretroviral Therapy (HAART) [7].

Anaemia at the time of HAART initiation has been associated with HIV disease progression and mortality [6, 8–12]. O'Brien et al. [12] in a recent study found that moderate and severe anaemia were associated with an increased mortality among Tanzanian women with HIV. This was true after controlling for potential confounders including CD4 cell count, clinical stage, and body mass index. As HIV disease severity progresses, the likelihood of developing anaemia increases [6], thus impacting negatively the quality of life of HIV patients. A study in 2009 by Obirikorang and Yeboah showed that the association between anaemia and mortality is causal and anaemia acts as a surrogate marker of the underlying disease, HIV [13]. In the developing world, several other etiologic factors such as micronutrient deficiencies and blood loss from intestinal opportunistic disease may also be involved in the development of HIV-associated anaemia [14].

So far, data on the levels of various markers of iron status in HIV are limited and in other instances are contradicting. High plasma ferritin concentrations have been found among HIV-infected patients [15–17] while other studies have reported low ferritin concentrations [18, 19]. Treatment regimens in the developed world may include erythropoietin; the high cost of this however restricts its use in resource-limited settings [20]. Studies from Europe and North America have shown that HAART can be an effective treatment for anaemia of HIV infection [21–23]. Whether the same holds true in Ghana and Africa, where comorbidities such as micronutrient deficiencies, malaria, TB, and parasitic infections are common, remains to be shown.

In this study, we determined the prevalence of anaemia in a cohort of HIV patients in a developing country and measured markers of iron homeostasis in relation to anaemia, disease progression, and HAART status.

## 2. Methodology

### 2.1. Study Design/Site

This comparative cross-sectional study was carried out at the Sexually Transmitted Infections (STI) clinic of the Tamale Teaching Hospital from July 2013 to December 2013. The hospital is located in the Tamale Metropolis of the Northern Region of Ghana. The hospital was established to serve as a medical referral centre for the Northern, Upper East, Upper West, and parts of the Brong Ahafo Regions of Ghana and also for neighbouring countries: La Cote d'Ivoire, Burkina Faso, and Togo.

### 2.2. Ethical Consent

The study was approved by the Committee on Human Research, Publication and Ethics (CHRPE) of the School of Medical Sciences (SMS), Kwame Nkrumah University of Science and Technology (KNUST), and the Department of Research and Monitoring, Tamale Teaching Hospital. Participation was voluntary and written informed consent was obtained from each participant.

### 2.3. Study Population

At the time of first presentation at the hospital, all patients (both HAART-naive and On-HAART) have a “baseline visit” for a structured interview and laboratory examinations, after which they come for scheduled visits, as requested by the attending physician. Three hundred and nineteen participants were recruited for the study, which consisted of 219 patients on HAART (designated On-HAART) and 100 HAART-naive patients (designated HAART-naive). Patients were included in the study if they were diagnosed and confirmed HIV-positive, aged ≥ 18 years, followed up by the Tamale Teaching Hospital STI clinic, on HAART for at least three (3) months, and with good adherence to therapy. Good adherence was defined as missing <2 doses of 30 doses or <3 doses of 60 doses [24]. Patients who were pregnant and those with inflammation, defined as a CRP > 8.2 mg/L [25], were excluded.

### 2.4. Data Collection and Laboratory Methods

An interview-based questionnaire was used to gather data on sociodemography. Clinical history was obtained from patient records.

Five mL of early morning venous blood was drawn from each participant; 2 mL was dispensed into a vacutainer tube containing ethylenediaminetetraacetic acid (EDTA) and 3 mL was dispensed into a serum separator tube (SST), allowed to clot, and then centrifuged at 3000 g for 5 minutes. Aliquots of the serum were stored at −80°C until assays were performed. Laboratory assays included CD4/CD3 lymphocyte counts by flow cytometry (BD FACSCOUNT, Becton Dickenson and Company, California, USA) and haemoglobin and red/white cell indices (Mindray BC 3000 Plus, Mindray Company, Shenzhen, China). Serum iron, ferritin, transferrin, and transferrin saturation (TSAT) were measured using the Flexor XL analyser from vital scientific. Serum CRP was also performed with a semiquantitative immune-chromatographic method, to guide in excluding a rise in serum ferritin due to acute inflammation [26].

### 2.5. Data Analysis and Statistics

Disease progression as indicated by CD4 count was Stage 1 (≥500 cells/mm^3^), Stage 2 (200–499 cells/mm^3^), and Stage 3 (<200 cells/mm^3^) as per recommendations of the Center for Disease Control (CDC) [27–29]. The World Health Organization/Association of Clinical Trial Group (WHO/ACTG) criteria were used to define mild (Hb 10.5–12.99 g/dL for men; 10.5–11.99 g/dL for women), moderate (Hb 8.0–10.49 g/dL), and severe (Hb < 8.0 g/dL) anaemia [30, 31]. Using the Mean Cell Volume (MCV), reference limits of 80–96 fL and Mean Cell Haemoglobin (MCH) reference limits of 27–32 pg as pointers, low MCV (<80 fL) was indicative of microcytosis, high MCV (>96 fL) was indicative of macrocytosis, and low MCH (<27 pg) indicates hypochromia [32]. Iron deficiency (ID) was defined as serum iron < 8.6 *μ*mol/L, ferritin < 15 *μ*g/L, and TSAT < 16%. Iron deficiency anaemia (IDA) was defined as Hb ≤ 10.5 g/dL, serum iron ≤ 8.6 *μ*mol/L, ferritin < 15 *μ*g/L, and TSAT < 16%. Iron overload was defined as serum iron > 30 *μ*mol/L, ferritin > 300 *μ*g/L, and TSAT > 55%.

Data are presented as mean ± standard deviation (SD) or *n* (%). Unpaired *t-*test was used to compare the means of all continuous variables. Categorical data were analysed using Fisher's exact test or Chi-square where applicable. Multiple variables were analysed using the one-way ANOVA. For all statistical comparisons, a *P* value of <0.05 was considered significant. Data were analysed using GraphPad Prism version 6.0 for windows (GraphPad software, San Diego, California, USA).

## 3. Results

Of the total, there were significantly more females than males on HAART (*P* = 0.0028) (Table 1).

On-HAART participants had significantly higher CD4/CD3 lymphocyte counts, Hb, haematocrit, MCV, MCH, RDW-SD, serum iron, ferritin, and transferrin saturation than when compared to their corresponding HAART-naive group (*P* < 0.05). WBC count (within normal limits of reference), serum transferrin, and TIBC were however higher amongst the HAART-naive individuals (*P* < 0.05) (Table 2).

Seventy-six participants had anaemia, representing 23.8% of the study population. When grouped based on haemoglobin concentration, participants with anaemia had a significantly lower CD4/CD3 lymphocyte count (*P* < 0.0001 each) with corresponding lower mean values of red cell indices (HCT, *P* < 0.0001; MCV, *P* < 0.0001; MCH, *P* < 0.0001; MCHC, *P* = 0.0017). Likewise, serum iron (*P* = 0.0019), ferritin (*P* = 0.0021), and TSAT (*P* = 0.0002) were significantly lower in anaemic patients than in those without anaemia. Serum transferrin (*P* < 0.0001) and TIBC (*P* < 0.0001) were however higher in anaemic patients than in nonanaemic patients (Table 3).

There was a decreasing trend in mean values of haemoglobin and corresponding red cell indices from grade 1 to grade 3 anaemia. A similar trend was observed with serum iron, ferritin, and TSAT, but the reverse in the case of serum transferrin and TIBC (increased from grade 1 through grade 3). CD4/CD3 lymphocyte counts were lowest in grade 3 anaemia. There were individual differences between various parameters when compared within and across groups (Table 4).

In Table 5, participants are grouped according to the severity of disease using CD4. Immunological markers (CD4, *P* < 0.0001; CD3, *P* < 0.0001; WBC, *P* = 0.1597) and serum iron (*P* = 0.0072) decreased with severity of disease. Serum ferritin (*P* = 0.9022), transferrin (*P* = 0.0143), and TIBC (*P* = 0.0143) increased with severity of disease. There were individual differences between various parameters when compared within and across groups.

Characteristics of study participants, grouped according to iron status, are shown in Table 6. From the definitive criteria used, 76 (23.8%) participants fulfilled the criteria for anaemia, 86 (26.9%) for iron deficiency, 41 (12.8%) for iron deficiency anaemia, and 17 (5.3%) for iron overload. Significant linear trends were observed for HB, HCT, iron, and TSAT.

The frequency of anaemia was higher amongst participants not on HAART (OR 2.6 for grade 1 anaemia; OR 3.0 times for grade 3 anaemia (*P* < 0.05)) (Table 7).

## 4. Discussion

In this study, we determined the prevalence of anaemia in a cohort of HIV patients and measured markers of iron homeostasis in relation to anaemia, disease progression, and HAART. We showed that, in this population, HIV-associated anaemia is common and is associated with disease progression. The frequency of anaemia was higher amongst participants not on HAART.

In the present study, prevalence of anaemia in the total population was 23.8%. Our prevalence is similar to that observed in advanced countries [22, 33] but significantly lower than that observed by other researchers in neighbouring African countries [11, 34, 35]. In a recent study conducted in Tanzania, a prevalence of 77.4% was recorded and this was attributed to advanced immunodeficiency and the high prevalence of anaemia in the African subregion [11]. We however believe the observed prevalence of anaemia may be due to the high proportion of On-HAART individuals. In support, a study conducted in Tanzania showed that anaemia was far more frequent in HIV-infected adults than in their HIV-negative counterparts [36].

We found that anaemia was consistent with increasing disease progression of HIV as per recommendations of the Center for Disease Control (CDC) [27–29]. Similarly, anaemia at the time of HAART initiation has been associated with HIV disease progression and mortality [6, 8–12]. This association has been linked to an increasing viral burden as HIV disease progresses and may cause anaemia through increased cytokine-mediated myelosuppression. HAART on the other hand has been shown to significantly treat anaemia of HIV infection [21–23], thus reducing the incidence of anaemia in HIV. This is in line with our observation that the frequency of anaemia was higher amongst participants not on HAART. HAART in itself may have a protective effect on the development of anaemia, through reduced disease progression. Further evidence from a study conducted in Tanzania showed that haemoglobin level increased significantly at 2.5 g/dL over the first 12 months in patients who received HAART [11].

With the complexity of HIV disease, however, anaemia may be a surrogate marker of some other underlying condition, which may not be captured through categorization with CD4 lymphocyte counts and clinical AIDS diagnosis alone [33]. Furthermore, anaemia can be a feature of certain opportunistic diseases [37] and other etiologic factors including micronutrient deficiencies and blood loss from intestinal opportunistic disease [14, 37], a common feature in the developing world. This is consistent with our observation that haemoglobin concentration, serum iron, ferritin, and TSAT decreased from grade 1 to grade 3 anaemia. Iron supplementation is however feared, as adverse effects of iron excess in HIV-infected individuals have been reported in industrialized countries [38]. This is similar to our observation that the mean levels of iron stores were within normal limits.

It is thought that the predominant cause of anaemia in the context of HIV is anaemia of inflammation (AI), also known as anaemia of chronic disease [1]. The observed WBC count was higher amongst the HAART-naive individuals, who had a higher prevalence and frequency of anaemia. It is noteworthy that although inflammation may play a role in the higher prevalence of anaemia amongst the HAART-naive individuals, WBC values were within normal reference limits and we measured CRP to rule out all participants who had active inflammation. Furthermore, immunological markers decreased significantly with increasing severity of disease (Table 5). In this instance, anaemia of chronic disease may be a more appropriate term for our study participants rather than anaemia of chronic inflammation.

Serum iron, ferritin, and TSAT were significantly lower in HIV patients with anaemia than in those without anaemia. Iron supplementation has been shown to reduce anaemia in HIV patients, without adverse effects on HCV coinfection or plasma HIV-RNA [37]. Notwithstanding, it has been suggested that care must be taken in iron supplementation, to prevent adverse effects of iron excess in HIV-infected individuals [38]. The present study is in agreement with this suggestion as the mean levels of these markers of iron stores for all participants (with HIV) were within normal limits. Thus, in this case, iron deficiency may not be a key player in anaemia in HIV.

In this study, we used haemoglobin as measure of anaemia. Haemoglobin measurement plays an important role in the basic management of HIV disease in West Africa [39] and could be measured easily where resources for more sophisticated laboratory markers are not available. For better monitoring and intervention, regular measurements could be of great help.

## 5. Conclusion

In this population, HIV-associated anaemia is common. Anaemia was more prevalent in severe disease forms and the frequency of anaemia was higher in participants yet to initiate HAART. High serum ferritin, transferrin, and TIBC in severe disease forms suggest low iron distribution in HIV infection and that HIV infection does not adversely influence tissue iron availability. The present study confirms that HIV itself is the most important cause of anaemia and that iron deficiency is of less importance. The treatment of HIV should be a priority in management strategies compared to iron supplementation.

## Figures and Tables

**Table 1:** Sociodemographic characteristic of the study population.

Parameter	Total (*n* = 319)	On-HAART (*n* = 219)	HAART-naive (*n* = 100)	*P* value
Gender *n* (%)				
Female	217 (68.0)	161 (73.5)	56 (56.0)	0.0028
Male	102 (32.0)	58 (26.5)	44 (44.0)	
Age (yrs)				
Mean ± SD	38.89 ± 9.91	39.93 ± 9.72	37.91 ± 9.53	0.0842
18–24 *n* (%)	25 (7.8)	11 (5.0)	14 (14.0)	0.0076
25–34 *n* (%)	96 (30.1)	62 (28.3)	34 (34.0)	
35–44 *n* (%)	108 (33.9)	83 (37.9)	25 (25.0)	
45–54 *n* (%)	55 (17.2)	42 (19.1)	13 (13.0)	
>54 *n* (%)	35 (11.0)	21 (9.6)	14 (14.0)	
Marital status *n* (%)				
Married	198 (62.1)	142 (64.8)	56 (56.0)	0.1375
Single	121 (37.9)	77 (35.2)	44 (44.0)	
Educational level *n* (%)				
No education	106 (33.2)	76 (34.7)	30 (30.0)	
Basic education	92 (28.8)	69 (31.5)	23 (23.0)	
SSS	65 (20.4)	39 (17.8)	26 (26.0)	0.1432
Tertiary	56 (17.6)	35 (16.0)	21 (21.0)	
Employment status *n* (%)				
Formal	73 (22.9)	47 (21.5)	26 (26.0)	0.0591
Informal	175 (54.9)	129 (58.9)	46 (46.0)	
Unemployed	71 (22.3)	43 (19.6)	28 (28.0)	

Data are presented as mean ± SD or *n* (%). *P* values represent comparisons between On-HAART and HAART-naïve participants. *P* values < 0.05 were considered significant. HAART = Highly Active Antiretroviral Therapy.

**Table 2:** Immunological, haematological, and biochemical profiles of the study population stratified by HAART status.

Parameter	Total (*n* = 319)	On-HAART (*n* = 219)	HAART-naive (*n* = 100)	*P* value
Immunological profile (mean ± SD)				
CD4 (cells/mm^3^)	426.40 ± 191.40	475.40 ± 229.50	207.10 ± 102.20	<0.0001
CD3 (cells/mm^3^)	1329.00 ± 513.70	1394.00 ± 403.9	1036.00 ± 377.2	0.0007
CD4/CD3	0.31 ± 0.12	0.34 ± 0.12	0.20 ± 0.56	<0.0001
WBC (10^3^/*μ*L)	5.23 ± 1.86	5.09 ± 1.81	5.88 ± 1.70	0.0151
Haematological profile (mean ± SD)				
Hb (g/dL)	11.65 ± 1.70	11.89 ± 1.56	10.59 ± 1.88	<0.0001
RBC (10^6^/*μ*L)	3.86 ± 0.58	3.84 ± 0.58	3.93 ± 0.62	0.3576
HCT (%)	35.01 ± 4.76	35.67 ± 4.41	32.04 ± 5.18	<0.0001
MCV (fL)	91.82 ± 11.73	97.99 ± 10.96	82.09 ± 10.08	<0.0001
MCH (pg)	31.79 ± 5.14	32.67 ± 4.97	27.87 ± 3.98	<0.0001
MCHC (g/dL)	34.62 ± 2.83	34.75 ± 2.78	34.03 ± 2.99	0.1433
RDW-CV	15.17 ± 1.76	14.96 ± 1.57	16.13 ± 2.25	0.0001
RDW-SD	52.20 ± 7.28	52.82 ± 7.41	49.40 ± 5.99	0.0068
PLT (10^3^/*μ*L)	242.3 ± 78.75	240.50 ± 71.32	250.40 ± 106.60	0.4756
Biochemical profile (mean ± SD)				
Iron (*μ*mol/L)	13.63 ± 11.73	14.51 ± 12.40	9.70 ± 3.94	0.0187
Ferritin (*μ*g/L)	255.00 ± 51.48	265.20 ± 89.96	238.10 ± 57.45	0.0691
Transferrin (mg/dL)	203.90 ± 36.81	199.60 ± 30.28	223.20 ± 54.05	0.0002
TIBC (*μ*g/dL)	259.00 ± 46.75	253.50 ± 38.45	283.50 ± 68.64	0.0002
% TSAT	30.82 ± 27.08	33.00 ± 18.57	21.06 ± 10.85	0.0114

Data are presented as mean ± SD. *P* values represent comparisons between On-HAART and HAART-naïve participants. *P* values < 0.05 were considered significant. WBC = White Blood Cells, Hb = haemoglobin, RBC = Red Blood Cells, HCT = haematocrit, MCV = Mean Cell Volume, MCH = Mean Cell Haemoglobin, MCHC = Mean Cell Haemoglobin Concentration, RDW-CV = Red Cell Distribution Width-Cumulative Variance, RDW-SD = Red Cell Distribution Width-Standard Deviation, PLT = platelets, TIBC = Total Iron Binding Capacity, TSAT = transferrin saturation, and HAART = Highly Active Antiretroviral Therapy.

**Table 3:** Immunological, haematological, and biochemical profiles of the study population stratified by anaemia status.

Parameter	Anaemia (*n* = 76)	No anaemia (*n* = 243)	*P* value
Age (yrs)	36.87 ± 11.25	39.51 ± 9.40	0.0924
Immunological profile (mean ± SD)			
CD4 (cells/mm^3^)	264.50 ± 98.40	476.80 ± 164.30	<0.0001
CD3 (cells/mm^3^)	1029.00 ± 485.20	1422.00 ± 559.90	<0.0001
CD4/CD3	0.23 ± 0.04	0.34 ± 0.13	<0.0001
WBC (10^3^/*μ*L)	5.06 ± 2.01	5.29 ± 1.18	0.4585
Haematological profile (mean ± SD)			
Hb (g/dL)	9.32 ± 1.17	12.38 ± 1.06	<0.0001
RBC (10^6^/*μ*L)	3.43 ± 0.52	3.99 ± 0.54	0.5029
HCT (%)	28.89 ± 3.56	36.91 ± 3.24	<0.0001
MCV (fL)	85.41 ± 12.13	93.81 ± 10.89	<0.0001
MCH (pg)	28.39 ± 5.30	32.85 ± 4.62	<0.0001
MCHC (g/dL)	33.24 ± 3.21	35.05 ± 2.56	0.0017
RDW-CV	16.61 ± 2.16	14.73 ± 1.34	<0.0001
RDW-SD	52.59 ± 8.49	52.07 ± 6.88	0.3865
PLT (10^3^/*μ*L)	251.50 ± 91.12	239.40 ± 74.56	0.3357
Biochemical profile (mean ± SD)			
Iron (*μ*mol/L)	8.93 ± 3.99	15.09 ± 6.52	0.0019
Ferritin (*μ*g/L)	228.70 ± 56.48	270.10 ± 39.21	0.0021
Transferrin (mg/dL)	229.00 ± 55.13	196.10 ± 24.23	<0.0001
TIBC (*μ*g/dL)	290.80 ± 70.02	249.10 ± 30.77	<0.0001
% TSAT	18.87 ± 8.15	35.54 ± 15.83	0.0002

Data are presented as mean ± SD. *P* values < 0.05 were considered significant. WBC = White Blood Cells, Hb = haemoglobin, RBC = Red Blood Cells, HCT = haematocrit, MCV = Mean Cell Volume, MCH = Mean Cell Haemoglobin, MCHC = Mean Cell Haemoglobin Concentration, RDW-CV = Red Cell Distribution Width-Cumulative Variance, RDW-SD = Red Cell Distribution Width-Standard Deviation, PLT = platelets, TIBC = Total Iron Binding Capacity, TSAT = transferrin saturation, and HAART = Highly Active Antiretroviral Therapy.

**Table 4:** Immunological, haematological, and biochemical profiles of the study population stratified by grade of anaemia.

Parameter	Grade 1 (*n* = 41)	Grade 2 (*n* = 19)	Grade 3 (*n* = 16)	*P* value
Age (yrs)	35.79 ± 10.64	42.36 ± 14.66	33.75 ± 5.85	0.0591
Immunological profile (mean ± SD)				
CD4 (cells/mm^3^)	271.50 ± 121.10	327.60 ± 135.40	148.90 ± 71.13^*∗∗∗*††††^	<0.0001
CD3 (cells/mm^3^)	1027.00 ± 485.7	1050.00 ± 465.21	1011.00 ± 407.20	0.0008
CD4/CD3	0.24 ± 0.14	0.27 ± 0.07	0.14 ± 0.05^*∗∗*††††^	<0.0001
WBC (10^3^/*μ*L)	4.78 ± 1.87	5.58 ± 2.22	5.58 ± 1.67	0.4141
Haematological profile (mean ± SD)				
Hb (g/dL)	10.03 ± 0.26	8.92 ± 0.48^*∗∗∗∗*^	6.95 ± 0.54^*∗∗∗∗*††††^	<0.0001
RBC (10^6^/*μ*L)	3.53 ± 0.43	3.50 ± 0.61	2.95 ± 0.49^*∗∗∗*††^	<0.0001
HCT (%)	30.88 ± 1.76	27.68 ± 2.32^*∗∗∗∗*^	22.39 ± 1.13^*∗∗∗∗*††††^	<0.0001
MCV (fL)	88.68 ± 10.99	81.26 ± 13.29^*∗*^	77.60 ± 10.94^*∗∗*^	<0.0001
MCH (pg)	29.78 ± 4.88	27.34 ± 5.59	24.11 ± 4.44^*∗∗∗*^	<0.0001
MCHC (g/dL)	33.61 ± 2.99	33.72 ± 3.89	31.03 ± 2.41^*∗∗*†^	<0.0001
RDW-CV	15.90 ± 1.67	17.01 ± 2.07^*∗*^	18.95 ± 2.55^*∗∗∗∗*†^	<0.0001
RDW-SD	52.69 ± 8.64	51.21 ± 7.38	54.11 ± 10.01	0.8142
PLT (10^3^/*μ*L)	237.90 ± 79.93	275.40 ± 105.30	274.90 ± 99.49	0.3053
Biochemical profile (mean ± SD)				
Iron (*μ*mol/L)	10.69 ± 4.81	6.46 ± 2.41^*∗∗∗*^	5.06 ± 2.75^**∗∗∗∗**^	0.0041
Ferritin (*μ*g/L)	216.1 ± 38.65	196.80 ± 75.50	181.00 ± 83.31^*∗*^	0.2346
Transferrin (mg/dL)	206.70 ± 34.25	234.30 ± 53.28^*∗*^	313.80 ± 46.19^*∗∗∗∗*††††^	0.0654
TIBC (*μ*g/dL)	262.50 ± 43.49	297.50 ± 66.66^*∗*^	398.50 ± 58.66^*∗∗∗∗*††††^	0.0654
% TSAT	23.38 ± 9.12	13.24 ± 4.12^*∗∗∗∗*^	8.00 ± 4.78^*∗∗∗∗*††^	0.0207

Data are presented as mean ± SD. *P* values were obtained using one-way ANOVA of all variables. *∗* represents comparisons with grade 1. † represents comparisons with grade 2. *∗*/† = <0.05. *∗∗*/†† = <0.01. *∗∗∗* = <0.001. *∗∗∗∗*/†††† = <0.0001. *P* values < 0.05 were considered significant. WBC = White Blood Cells, Hb = haemoglobin, RBC = Red Blood Cells, HCT = haematocrit, MCV = Mean Cell Volume, MCH = Mean Cell Haemoglobin, MCHC = Mean Cell Haemoglobin Concentration, RDW-CV = Red Cell Distribution Width-Cumulative Variance, RDW-SD = Red Cell Distribution Width-Standard Deviation, PLT = platelets, TIBC = Total Iron Binding Capacity, TSAT = transferrin saturation, and HAART = Highly Active Antiretroviral Therapy.

**Table 5:** Immunological, haematological, and biochemical profiles of the study population stratified by disease stage.

Parameter	CD4^+^ lymphocyte count, cell/mm^3^			
	Stage 1 (≥500)	Stage 2 (200–499)	Stage 3 (<200)	*P* value
Age (yrs)	38.04 ± 9.49	40.00 ± 9.46	38.24 ± 11.13	0.3871
Immunological profile				
CD3 (cell/mm^3^)	1775.00 ± 550.40	1273.00 ± 484.20^**∗**^	810.70 ± 310.70^**∗**†^	<0.0001
CD4/CD3	0.43 ± 0.09	0.32 ± 0.12^**∗**^	0.14 ± 0.06^**∗**†^	<0.0001
WBC (10^3^/*μ*L)	5.56 ± 1.65	5.12 ± 1.89	4.97 ± 2.04	0.1597
Haematological profile				
Hb (g/dL)	12.28 ± 1.37	11.92 ± 1.38	10.35 ± 1.90^**∗**†^	<0.0001
RBC (10^6^/*μ*L)	4.01 ± 0.55	3.85 ± 0.53	3.64 ± 0.65^**∗**^	0.0015
HCT (%)	36.67 ± 3.74	35.82 ± 4.08	31.42 ± 5.21^**∗**†^	<0.0001
MCV (fL)	92.49 ± 11.20	94.06 ± 11.67	87.27 ± 11.48^**∗**†^	0.0025
MCH (pg)	32.55 ± 5.48	32.41 ± 4.42	29.75 ± 5.30^**∗**†^	0.0027
MCHC (g/dL)	35.11 ± 3.64	34.54 ± 2.63	34.08 ± 3.29	0.1120
RDW-CV	14.64 ± 1.43	15.01 ± 1.55	16.16 ± 2.11^**∗**†^	<0.0001
RDW-SD	50.79 ± 6.42	52.74 ± 7.64	53.23 ± 7.61	0.1100
PLT (10^3^/*μ*L)	250.30 ± 68.68	238.80 ± 75.83	237.10 ± 95.21	0.5524
Biochemical profile				
Iron (*μ*mol/L)	14.73 ± 6.08	15.32 ± 12.08	9.38 ± 7.64^**∗**†^	0.0072
Ferritin (*μ*g/L)	260.80 ± 48.03	262.80 ± 42.05	266.00 ± 103.10	0.9022
Transferrin (mg/dL)	197.00 ± 25.38	202.50 ± 24.69	215.70 ± 58.18^**∗**^	0.0143
TIBC (*μ*g/dL)	250.20 ± 32.20	257.10 ± 31.35	273.90 ± 73.88	0.0143
% TSAT	33.60 ± 13.12	34.18 ± 15.26	21.59 ± 9.77^**∗**†^	0.0133

Data are presented as mean ± SD. *P* values < 0.05 were considered significant. *P* values represent one-way ANOVA of all three variables. *∗* represents comparisons with Stage 1. † represents comparisons with Stage 2. *∗*/† = <0.05. WBC = White Blood Cells, Hb = haemoglobin, RBC = Red Blood Cells, HCT = haematocrit, MCV = Mean Cell Volume, MCH = Mean Cell Haemoglobin, MCHC = Mean Cell Haemoglobin Concentration, RDW-CV = Red Cell Distribution Width-Cumulative Variance, RDW-SD = Red Cell Distribution Width-Standard Deviation, PLT = platelets, TIBC = Total Iron Binding Capacity, TSAT = transferrin saturation, and HAART = Highly Active Antiretroviral Therapy.

**Table 6:** Immunological, haematological, and biochemical profiles of the population under study stratified by iron status.

Parameter	Anaemia (*n* = 76)	Iron deficiency (*n* = 86)	Iron deficiency anaemia (*n* = 41)	Iron overload (*n* = 17)	*P* value	*P* value (trend)
Age	36.87 ± 11.25	38.23 ± 11.41	36.22 ± 10.37	38.85 ± 6.52	0.8002	0.7059
CD4 (cells/mm^3^)	264.50 ± 84.00	322.20 ± 102.80	233.10 ± 103.2	429.50 ± 124.1	0.211	0.1693
CD3 (cells/mm^3^)	1029.00 ± 425.20	1154.00 ± 401.00	1021.00 ± 426.1	1478.00 ± 452.10	0.1263	0.0519
CD4/CD3	0.23 ± 0.07	0.25 ± 0.07	0.19 ± 0.04	0.29 ± 0.09	0.2379	0.4632
WBC (10^3^/*μ*L)	5.06 ± 2.00	5.31 ± 0.29	5.57 ± 1.39	4.72 ± 1.20	0.6036	0.7046
Haematological profile						
Hb (g/dL)	9.32 ± 1.17	10.73 ± 2.19	8.89 ± 1.35	11.75 ± 1.11	<0.0001	0.0008
RBC (10^6^/*μ*L)	3.43 ± 0.52	3.86 ± 0.72	3.45 ± 0.56	3.79 ± 0.58	0.0016	0.2581
HCT (%)	28.89 ± 3.56	32.66 ± 6.13	27.60 ± 3.80	34.29 ± 3.46	<0.0001	0.0155
MCV (fL)	85.41 ± 12.13	85.70 ± 12.80	81.35 ± 12.36	92.12 ± 14.50	0.0954	0.1948
MCH (pg)	28.39 ± 5.30	29.18 ± 5.39	27.18 ± 5.54	31.72 ± 4.96	0.0785	0.1214
MCHC (g/dL)	33.24 ± 3.21	34.05 ± 3.04	33.36 ± 3.37	34.51 ± 1.15	0.3722	0.2879
RDW-CV	16.61 ± 2.16	15.76 ± 2.18	16.76 ± 2.08	14.98 ± 1.47	0.0161	0.0579
RDW-SD	52.59 ± 8.49	50.98 ± 6.86	50.66 ± 7.52	51.02 ± 7.05	0.6319	0.4892
PLT (10^3^/*μ*L)	251.50 ± 91.12	252.80 ± 95.10	270.60 ± 88.36	220.30 ± 97.97	0.461	0.3957
Biochemical profile						
Iron (*μ*mol/L)	8.93 ± 2.98	4.60 ± 1.82	4.01 ± 2.07	49.47 ± 19.94	<0.0001	<0.0001
Ferritin (*μ*g/L)	229.00 ± 55.13	223.80 ± 53.00	248.90 ± 62.32	193.10 ± 16.69	0.0215	0.1106
Transferrin (mg/dL)	290.80 ± 70.02	284.20 ± 67.31	316.10 ± 79.14	245.20 ± 21.20	0.0215	0.1106
TIBC (*μ*g/dL)	18.87 ± 7.49	9.60 ± 2.96	7.71 ± 2.24	112.80 ± 47.94	<0.0001	<0.0001

*P* values < 0.05 were considered significant. *P* values represent one-way ANOVA of all variables. WBC = White Blood Cells, Hb = haemoglobin, RBC = Red Blood Cells, HCT = haematocrit, MCV = Mean Cell Volume, MCH = Mean Cell Haemoglobin, MCHC = Mean Cell Haemoglobin Concentration, RDW-CV = Red Cell Distribution Width-Cumulative Variance, RDW-SD = Red Cell Distribution Width-Standard Deviation, PLT = platelets, TIBC = Total Iron Binding Capacity, TSAT = transferrin saturation, and HAART = Highly Active Antiretroviral Therapy.

**Table 7:** Anaemia and associated risks, amongst the study population.

Parameter	On-HAART (*n* = 219)	HAART-naive (*n* = 100)	OR (95% CI)	*P* value
WHO/ACTG				
Grade 1 *n* (%)	20 (9.1)	21 (21.0)	2.6 (1.3595 to 5.1458)	0.0042
Grade 2 *n* (%)	13 (5.9)	6 (6.0)	1.0 (0.373 to 2.743)	0.9821
Grade 3 *n* (%)	7 (3.2)	9 (9.0)	3.0 (1.082 to 8.289)	0.0346

WHO/ACTG = World Health Organization/AIDS Clinical Trial Group; OR = Odds Ratio, and HAART = Highly Active Antiretroviral Therapy. Reference = On-HAART.
